# In silico prediction of B cell epitopes and experimental validation on wheat allergens

**DOI:** 10.1186/2045-7022-4-S2-O4

**Published:** 2014-03-17

**Authors:** Sandra Denery-Papini, Virginie Lollier, Hamza Mameri, Manon Pietri, Colette Larre, Jean-Charles Gaudin, Olivier Tranquet, Martine Drouet, Evelyne Paty, Anne-Marie Jonathan, Etienne Beaudouin, Anne Denise Moneret-Vautrin, Dominique Tessier

**Affiliations:** 1 INRA, UR1268 BIA, NANTES, France; 2 Angers University Hospital, General Allergology Unit, Angers, France; 3 Necker Hospital, Pneumology and allergology department, Paris, France; 4 Cochin Hospital, Occupational disease department, Paris, France; 5 Epinal Hospital, Allergology Unit, Epinal, France

## Background

B cell epitopes corresponding to interaction zones between proteins and immune system are a mean to explore intrinsic features of proteins linked to allergenicity. The project PREDEXPITOPE was funded by the French National Research Agency to develop a bioinformatics strategy for prediction of B cell epitopes of allergens. The objective was to distinguish epitopic from non epitopic areas on the basis of frequently used criteria of surface exposition. In silico prediction was confronted with experimental results on wheat allergens.

## Method

Epitope sequences and description were exported from Immune Epitope DataBase (IEDB). Epitopes were mapped to 61 3D structures, extracted from PDB site. A pipeline program computed relative surface exposure of amino acids for each PDB file. Data were loaded into R environment for statistical comparison between epitopic and non epitopic amino acids. B epitopes were searched on wheat allergens for which 3D structure could be known. Sera were obtained from patients with food allergy to wheat or baker's asthma.

## Results

On the basis of the 61 antigen structures no clear-cut classification of epitopic and non-epitopic values could be statistically achieved. The amino acids within epitopes are not distributed above any threshold for both relative surface accessibility and protrusion index. The wheat serpin was a good candidate to validate our bioinformatics conclusion. A 3D structural model was constructed and areas of strong surface accessibility could be selected. These areas did not correspond to those bound by patient's IgE.

## Conclusion

Our work did not deny the requisite for B-cell epitopes to be exposed on surface. However, it indicates that the level of surface exposure has no discriminatory ability. The difficulty in establishing a system of prediction lies in the heterogeneity of data on B-cell epitopes, which varies from a few amino acids actually in contact with the antibodies to shorter or longer peptides, able to interact with these antibodies. The integration of experimental context or epidemiological aspects in epitope data bases may improve such a prediction.
